# Effects and safety of Huatan Qushi Huoxue granules on obesity-associated non-alcoholic fatty liver disease: a multicenter, randomized, double-blind, placebo-controlled trial

**DOI:** 10.3389/fphar.2026.1899068

**Published:** 2026-08-25

**Authors:** Lihui Zhang, Sutong Liu, Qing Zhao, Xiaoyan Liu, Xuehua Sun, Fenping Li, Miaoqing Ye, Minghao Liu, Lei Gao, Wenxia Zhao

**Affiliations:** 1 Hepatology (Gastroenterology) Regional Medical Center, The First Affiliated Hospital of Henan University of Chinese Medicine, Zhengzhou, Henan, China; 2 Collaborative Innovation Center of Prevention and Treatment of Major Diseases by Chinese and Western Medicine, Zhengzhou, Henan, China; 3 Department of Hepatology, Shuguang Hospital Affiliated to Shanghai University of Traditional Chinese Medicine, Shanghai, China; 4 Department of Hepatology, Shaanxi Provincial Hospital of Traditional Chinese Medicine, Xi’an, Shaanxi, China; 5 School of Basic Medical Sciences, Guangzhou University of Chinese Medicine, Guangzhou, Guangdong, China

**Keywords:** Huatan Qushi Huoxue granules, metabolomics, MRI-PDFF, non-alcoholic fatty liver disease, obesity, randomized controlled trial, traditional Chinese medicine

## Abstract

**Background:**

Non-alcoholic fatty liver disease (NAFLD) is a major global health issue strongly associated with obesity. Available treatments have significant limitations. The traditional Chinese medicine Huatan Qushi Huoxue (HTQSHX) granules represent a potential alternative therapeutic option. This study evaluates their effects and safety in patients with obesity-associated NAFLD.

**Methods:**

This multicenter, randomized, double-blind, placebo-controlled, parallel-group trial recruited 248 patients with obesity-associated NAFLD from three clinical sites. Participants were randomized 1:1 to receive HTQSHX granules or placebo granules for 12 weeks. The primary outcome was the absolute change in hepatic fat fraction from baseline to week 12, as measured by MRI-proton density fat fraction (MRI-PDFF), which is recommended as a primary endpoint in early-phase NAFLD clinical trials for its precision and objectivity. Secondary outcomes included the proportion of patients achieving ≥30% relative reduction in MRI-PDFF-assessed hepatic fat fraction, serum liver enzyme indicators, glucose and lipid metabolism parameters, non-alcoholic steatohepatitis-related serum biomarkers, anthropometric measures, and serum metabolomics analysis.

**Results:**

Among 228 participants included in the modified intention-to-treat (mITT) analysis, the median hepatic fat fraction decreased from 16.15% at baseline to 12.12% in the HTQSHX group (median change: 3.27%), and from 15.64% to 14.95% in the placebo group (median change: 0.96%). The between-group difference in absolute change was statistically significant (−2.32%; 95% CI, −4.50 to −0.23; *P* = 0.029). The proportion of patients achieving a ≥30% relative reduction in hepatic fat fraction was significantly higher in the HTQSHX group than in the placebo group (41.23% vs. 20.18%; *P* < 0.001). The HTQSHX group also showed significantly greater improvements in serum alanine aminotransferase (*P* = 0.048), aspartate aminotransferase (*P* = 0.033), free fatty acids (*P* = 0.026), cytokeratin-18 fragment M30 (*P* < 0.001), soluble triggering receptor expressed on myeloid cells 2 (*P* < 0.001) and anthropometric parameters (body mass index, waist-to-hip ratio, body fat percentage). Metabolomics analysis of 30 HTQSHX-treated participants revealed that the formula restored multiple phospholipid levels and reduced markers of oxidative stress and mitochondrial dysfunction, with enrichment in fatty acid and sphingolipid metabolism pathways.

**Conclusion:**

HTQSHX granules may represent a potentially beneficial therapeutic option for obesity-associated NAFLD, with observed effects including significant reduction in hepatic fat fraction and modulation of lipid metabolism and oxidative stress, alongside an acceptable safety profile. These findings warrant further investigation in longer-term studies.

**Clinical Trial Registration:**

https://www.chictr.org.cn/, identifier ChiCTR2200060901.

## Introduction

Non-alcoholic fatty liver disease (NAFLD) is a chronic progressive liver disease caused by overnutrition and insulin resistance in genetically susceptible individuals (Bugianesi and Petta, 2022). Its disease spectrum includes non-alcoholic fatty liver, non-alcoholic steatohepatitis (NASH), and related fibrosis and cirrhosis (Powell et al., 2021). The global prevalence rate of NAFLD is 30.05% (Younossi et al., 2023a). A meta-analysis based on 21 Asian cohorts, including Chinese populations, demonstrated that the risk of developing NAFLD in obese individuals was 3.53 times higher than that in individuals with normal BMI. Furthermore, a significant dose-response relationship was observed between BMI and NAFLD risk, with each 1 kg/m^2^ increase in BMI associated with a 20% increase in NAFLD risk (Li et al., 2016). NAFLD can lead to a series of adverse intrahepatic and extrahepatic outcomes (Zhang et al., 2025) and is a leading cause of liver-related mortality worldwide (Younossi, 2019). The clinical, economic, and quality-of-life burdens imposed by NAFLD are increasingly severe globally, underscoring the urgent need to develop global strategies to mitigate the negative impact of NAFLD on individuals and society (Do et al., 2025). It should be noted that the nomenclature for fatty liver disease is evolving. This trial was designed and conducted using the “NAFLD” framework, consistent with the Chinese guideline in effect at the time of its initiation (2018). Recent guidelines have adopted the term “metabolic dysfunction-associated fatty liver disease (MAFLD)” (Fan et al., 2024) to better reflect the metabolic pathogenesis. We retain the term NAFLD herein to ensure consistency with the study protocol and diagnostic criteria under which participants were enrolled.

Weight reduction through lifestyle modifications is an effective approach to reduce the disease burden of NAFLD (Younossi et al., 2023b). However, due to the substantial heterogeneity of NAFLD (Arrese et al., 2021), the benefits of weight loss may vary among patients with different characteristics (Stine et al., 2023). Resmetirom and semaglutide have successively received approval from the U.S. Food and Drug Administration for the treatment of patients with NASH accompanied by stage 2 or 3 liver fibrosis or histologically confirmed NASH (Keam, 2024; Sanyal et al., 2025). Nevertheless, key clinical issues for resmetirom, such as optimal treatment duration and discontinuation criteria, have not yet been clearly defined (The Lancet Gastroenterology Hepatology, 2024). On the other hand, semaglutide also has certain limitations in clinical application: it is associated with a high incidence of gastrointestinal adverse reactions (Loomba et al., 2023), long-term safety evidence still requires supplementation (Newsome et al., 2021), and weight rebound after discontinuation is relatively common (Xiao et al., 2024). The development of non-invasive imaging techniques is pivotal for advancing therapeutic evaluation in NAFLD. Although liver biopsy remains the histological reference standard, its clinical utility in serial monitoring and multicenter trials is limited by invasiveness, sampling variability, high cost, and risk of complications (Dunn et al., 2023). Conventional modalities such as ultrasound and computed tomography only provide semi-quantitative or qualitative assessment of hepatic steatosis, with insufficient sensitivity to detect moderate short-term changes in hepatic fat content. By contrast, MRI-derived proton density fat fraction (MRI-PDFF) has been established as the reference standard for accurate, non-invasive quantification of hepatic steatosis, a position endorsed by the 2021 updated EASL Clinical Practice Guidelines (2021). Owing to its excellent precision, high reproducibility, and ability to capture small longitudinal changes, MRI-PDFF is well validated as a reliable surrogate endpoint and is increasingly adopted as the primary efficacy endpoint in early-phase clinical trials of anti-steatotic agents (Tamaki et al., 2022a). Furthermore, a ≥30% relative reduction in MRI-PDFF-measured hepatic fat fraction is widely recognized as a clinically meaningful threshold, as it has been consistently associated with improvements in NAFLD activity score and histological fibrosis regression (Tamaki et al., 2022b). Given the 12-week intervention duration and multicenter design of this trial, MRI-PDFF was selected as the optimal primary outcome measure to reliably quantify changes in hepatic steatosis, while avoiding the practical and ethical limitations of invasive biopsy. Rooted in a profound understanding of NAFLD pathogenesis, traditional Chinese medicine (TCM) adopts a multi-target, holistic therapeutic approach that offers potential advantages for NAFLD management (Lei et al., 2025). According to TCM theory, the core pathogenesis of this disease is attributed to “phlegm-dampness and blood stasis obstructing the liver collaterals”, forming the theoretical rationale for the HTQSHX formulation (Liu et al., 2024). The Huatan Qushi Huoxue (HTQSHX) granules, developed on the basis of this pathogenesis, embody the core TCM therapeutic strategy for NAFLD. A previous multicenter, randomized clinical trial demonstrated that a modified regimen centered on the HTQSHX formula significantly improved serum alanine aminotransferase (ALT) levels, body mass index (BMI), and liver-to-spleen CT ratio in patients with non-alcoholic steatohepatitis (NASH) compared to polyene phosphatidylcholine capsules (Zhao et al., 2012). Experimental studies have shown that HTQSHX granules can alleviate mitochondrial damage, significantly reduce Fe^2+^ and hepatic iron content, and mitigate iron deposition (Liu et al., 2025). The pharmacological plausibility of this formulation is further supported by evidence that several of its bioactive plant metabolites - including quercetin, oleanolic acid, tanshinone IIA, and luteolin - have been demonstrated in clinical and preclinical studies to ameliorate hepatic steatosis, reduce lipotoxicity, and improve insulin resistance via modulation of lipid metabolism, oxidative stress, and inflammatory pathways (Li et al., 2024; Pi et al., 2024; Xue et al., 2021; Zhang et al., 2024). The present study focuses specifically on obesity-associated NAFLD, as obesity is well established as the strongest modifiable risk factor for NAFLD onset and histological progression (Rodriguez and Hartmann, 2025). While NAFLD also occurs in non-obese individuals, its underlying pathophysiology differs notably, with genetic susceptibility and visceral adipose dysfunction playing a more prominent role in this phenotype. The HTQSHX formulation is designed to resolve “phlegm-dampness and blood stasis”, pathological patterns closely linked to obesity-related metabolic disturbances, and may therefore exert more targeted therapeutic effects in this specific patient population. However, no head-to-head comparative clinical data are currently available regarding the therapeutic effects of HTQSHX in obese versus non-obese NAFLD patients. Despite these promising findings, previous studies on HTQSHX granules have notable limitations. The prior clinical trial, while randomized and multicenter, used liver-to-spleen CT ratio rather than the currently recommended quantitative imaging biomarker MRI-PDFF as its primary endpoint. Experimental studies have provided mechanistic insights, but these were limited to *in vitro* and animal models without clinical correlation. Furthermore, no study has specifically evaluated HTQSHX granules in the obesity-associated NAFLD population using a rigorously designed, double-blind, placebo-controlled paradigm with an objective quantitative endpoint. To address these gaps, the present trial was designed as a multicenter, randomized, double-blind, placebo-controlled study employing MRI-PDFF as the primary outcome measure to provide high-level evidence on the effects and safety of HTQSHX granules specifically in patients with obesity-associated NAFLD.

## Materials and methods

### Ethnopharmacological reporting and quality control compliance

This study fully adheres to the Four Pillars of Best Practice in Ethnopharmacology and the ConPhyMP consensus guidelines for phytochemical characterization of medicinal plant extracts, as required by the journal. (1) Traditional use rationale: The HTQSHX formula is developed based on the core TCM pathogenesis theory of “phlegm-dampness and blood stasis obstructing liver collaterals” for NAFLD. Its clinical application basis and prior clinical efficacy evidence are detailed in the Introduction (Zhao et al., 2012). (2) Botanical characterization: All eight constituent botanical drugs comply with the quality standards of the *Chinese Pharmacopoeia*. Complete botanical nomenclature, including scientific names with authorities and family assignments, is provided in Table 1. (3) Ethical sourcing and regulatory status: All botanical drugs are sourced from standardized cultivated populations via licensed commercial suppliers of botanical drugs in China. None of the species are listed in the CITES appendices or classified as protected wild species under Chinese regulations. No wild-harvested materials were used in the production of the investigational product, and all sourcing practices comply with botanical drug administration regulations of China. (4) Chemical characterization: The chemical profile of HTQSHX granules is defined by HPLC fingerprinting and comprehensive qualitative analysis via LC-MS/MS, identifying 48 characteristic metabolites (Supplementary Material 3; Supplementary Figure S1). The completed ConPhyMP assessment checklist and full quality control report are provided as Supplementary Material 7. (5) Pharmacological evaluation: The therapeutic dosage used in this trial is derived from conventional clinical practice and validated by a prior multicenter clinical trial (Zhao et al., 2012). All investigational products were manufactured under GMP conditions with full batch release quality testing, ensuring consistent product quality and subject safety throughout the trial, in full compliance with ethical requirements for clinical trial interventions.

**TABLE 1:** Standard formulation of HTQSHX granules.

Pinyin name	Latin scientific name (authority and family)	Medicinal part	Amount (%)	TCM action
Zexie	*Alisma plantago-aquatica* L. (*Alismataceae*)	Rhizoma	24.78	Promoting diuresis and eliminating dampness, regulating qi and dissipating blood stasis
Danshen	*Salvia miltiorrhiza* Bunge (*Lamiaceae*)	Radix et Rhizoma	12.4	Invigorating blood circulation to resolve stasis, cooling blood and calming spirit
Yujin	*Curcuma wenyujin* Y.H. Chen & C. Ling (*Zingiberaceae*)	Radix Curcumae	12.4	Invigorating blood flow to alleviate pain, regulating qi and relieving depression
Haizao	*Sargassum pallidum* (Turner) C. Agardh (*Sargassaceae*)	Thallus	12.4	Resolving phlegm and dampness, softening hard masses
Juemingzi	*Senna tora* (L.) Roxb. (*Fabaceae*)	Semen	8.26	Clearing liver and improving vision
Shanzha	*Crataegus pinnatifida* Bunge (*Rosaceae*)	Fructus	12.4	Resolving food stagnation and dissipating blood stasis
Shuifeiji	*Silybum marianum* (L.) Gaertn. (*Asteraceae*)	Fructus	12.4	Clearing heat and detoxifying
Chaihu	*Bupleurum chinense* DC. (*Apiaceae*)	Radix	4.96	Soothing liver and regulating qi, acting as the guiding botanical drug

HTQSHX, Huatan Qushi Huoxue.

### Study design

This multicenter, randomized, double-blind, placebo-controlled trial was conducted across three clinical centers in China: the First Affiliated Hospital of Henan University of Chinese Medicine, Shuguang Hospital Affiliated to Shanghai University of Traditional Chinese Medicine, and Shaanxi Provincial Hospital of Chinese Medicine. A total of 248 participants were randomly assigned in a 1:1 ratio to receive either HTQSHX granules or a matching placebo. All participants provided written informed consent. The study protocol received approval from the Medical Ethics Committee of the First Affiliated Hospital of Henan University of Chinese Medicine (Approval No. 2022HL-038-01). The trial reporting adheres to the CONSORT guidelines (Schulz et al., 2010).

### Participants

Patients were included as follows: (1) Meeting the NAFLD diagnostic criteria (2018), which define “non-alcoholic” as alcohol consumption <30 g/day for men and <20 g/day for women, with a confirmed diagnosis of obesity requiring BMI ≥28 kg/m^2^ or waist circumference ≥85 cm (men)/80 cm (women); (2) Aged 18–65 years; (3) Serum ALT level ranging from 1 to 3×ULN; (4) Fasting glucose ≤7.0 mmol/L; (5) Provision of written informed consent; (6) No recent NAFLD/obesity drugs (or ≥4-week washout). The exclusion criteria were: (1) Presence of other liver diseases, including alcoholic liver disease, chronic viral hepatitis, drug-induced liver injury, autoimmune hepatitis, Wilson’s disease, and fatty liver secondary to total parenteral nutrition; (2) History of severe cardiac, cerebral, renal, or hematologic system diseases, or diagnosed psychiatric disorders; (3) Diagnosis of type 1 diabetes mellitus; (4) Current or historical diagnosis of any malignancy; (5) Pregnancy or lactation; (6) Concurrent participation in any other clinical trial. (7) Known allergy or hypersensitivity to any constituent botanical drug or excipient of the investigational products. Alcohol consumption status was verified via a standardized structured interview of drinking history at baseline screening, to confirm compliance with the non-alcoholic eligibility criterion.

### Randomization and blinding

The randomization and blinding procedures were conducted by the Research Centre for Evidence-Based Medicine of Traditional Chinese Medicine in Henan Province. Using R software (version 4.1.2; R Foundation for Statistical Computing), the centre generated a two-level randomization sequence. The first level assigned each sequential case number to either Group A or Group B, while the second level determined the specific treatment (HTQSHX granules or placebo) corresponding to each group. All study drugs were then packaged and labeled according to this pre-defined random code. Throughout the trial, all investigators and participants remained blinded to the group assignments. After database lock upon study completion, the first unblinding was performed by the center’s staff. This step revealed only the group allocation (A or B) for each case number to the statisticians to enable comparative analysis. Following the completion of all statistical analyses and the drafting of the summary report, the second unblinding took place at the clinical wrap-up meeting to disclose the actual treatment associated with each group. The randomization parameters were set as follows: total sample size (N = 248), number of centers (3), number of treatment groups (2), allocation ratio (1:1), block length (8), resulting in 31 blocks. To facilitate drug distribution and management, a unique drug kit numbering system was pre-defined for each center: Shaanxi Provincial Hospital of Traditional Chinese Medicine (kits 1-80), Shuguang Hospital Affiliated to Shanghai University of Traditional Chinese Medicine (kits 81-160), and The First Affiliated Hospital of Henan University of Chinese Medicine (kits 161-248). All study drugs were identical in appearance, packaging, and labeling. They were packaged and labeled strictly according to the pre-generated random code by Jiangsu Jiangyin Tianjiang Pharmaceutical Co., Ltd., who had no role in the trial conduct. Drug kits were then distributed to the respective centers based on the numbering system. Throughout the trial, investigators, participants, and outcome assessors remained blinded to the kit assignment.

### Intervention

Patients in the HTQSHX group received HTQSHX granules (12 g per bag), administered orally as 2 bags twice daily (total daily dose: 24 g) after being dissolved in approximately 100 mL of hot water. This dosage regimen was selected based on a prior multicenter randomized clinical trial using a modified regimen centered on the HTQSHX formula in patients with NASH (Zhao et al., 2012), in which the equivalent therapeutic dose demonstrated favorable efficacy and good tolerability. The dose also aligns with the conventional clinical dosage range of this formula in long-standing traditional Chinese medicine practice. The medication was taken 1 h after breakfast and dinner, for 6 consecutive days per week followed by a 1-day break, over a total treatment period of 12 weeks. The placebo group received granules matched to the HTQSHX granules in appearance, odor, and taste (12 g per bag), composed of maltodextrin, lactose, caramel pigment, lemon yellow, sunset yellow, and a bittering agent. The dosage regimen and administration method were identical to those used in the HTQSHX group. Both the HTQSHX and placebo granules were manufactured by Jiangsu Jiangyin Tianjiang Pharmaceutical Co., Ltd. (batch number: 2205302) under strict Good Manufacturing Practice (GMP) conditions. For HTQSHX granules, each of the eight constituent botanical drugs was individually extracted with purified water in accordance with the *Chinese Pharmacopoeia* standards for botanical drug formula granules. The extraction parameters were optimized for each botanical drug according to its pharmacopoeial monograph: for example, *Alisma plantago-aquatica* L. (Zexie) was extracted twice with 6–14 volumes of water, each for 0.5–2 h; *Sargassum pallidum* (Turner) C.Agardh (Haizao) was extracted once with 20–30 volumes of water for 0.5–2 h; *Salvia miltiorrhiza* Bunge (Danshen) was extracted twice with 6–12 volumes of water, each for 0.5–2.5 h; the remaining constituents were extracted under their respective pharmacopoeia-specified conditions. Each intermediate extract was quality-controlled against its corresponding pharmacopoeial marker compound specification prior to blending. The aqueous extracts from each botanical drug were separately filtered, concentrated under reduced pressure at 60 °C to the specified relative density, and then spray-dried into intermediate dry powders. The resulting powders were blended precisely according to the proportional formula specified in Table 1, mixed for 30 min to ensure uniformity, and passed through a 100-mesh sieve. The blended mixture was then processed into uniform 12–30 mesh granules via dry granulation, with a small amount of maltodextrin added as a processing excipient. Final product consistency and chemical profile were verified by HPLC fingerprinting and LC-MS/MS qualitative analysis, as detailed in Supplementary Material 3 (Supplementary Figure S1; Supplementary Table S1). The botanical composition of the HTQSHX formulation is detailed in Table 1. Concomitant medications used during the trial were recorded in the case report forms (CRFs). While necessary treatments for other diseases under clinical guidance were permitted, the use of hepatoprotective, anti-inflammatory, or lipid-lowering agents was prohibited throughout the trial period. All patients received standardized diet and exercise counseling tailored to local lifestyle habits for the duration of the study. Medication adherence was calculated for each participant based on the count of returned, unused granule sachets at each study visit. Adherence rate was defined as: (Number of sachets dispensed - Number of sachets returned)/Number of sachets dispensed × 100%.

### Serum metabolomics analysis

Serum samples (100 μL) were extracted with methanol/acetonitrile (1:1, v/v), sonicated, and centrifuged. The supernatant was dried and reconstituted. LC-MS/MS analysis was performed on a Waters ACQUITY UPLC system coupled to a timsTOF Pro mass spectrometer. Metabolites were identified using Compound Discoverer 3.3 against mzCloud, NIST, and HMDB databases. Differential metabolites were screened by fold change, *P* < 0.05, and VIP values from OPLS-DA, followed by KEGG enrichment analysis.

### Outcomes

The primary outcome was the absolute change in hepatic fat fraction, as measured by MRI-PDFF, from baseline to week 12. Secondary outcomes included: the proportion of patients achieving a ≥30% relative reduction in hepatic fat fraction (MRI-PDFF); Changes in serum liver enzymes: alanine aminotransferase (ALT) and aspartate aminotransferase (AST); Changes in glucose and lipid metabolism parameters: total cholesterol (TC), triglycerides (TG), high-density lipoprotein cholesterol (HDL-C), low-density lipoprotein cholesterol (LDL-C), free fatty acids (FFA), fasting blood glucose (FBG), fasting insulin (FINS), and homeostasis model assessment of insulin resistance (HOMA-IR); Changes in NASH-related biomarkers: cytokeratin-18 fragment M30 (CK18-M30) and soluble triggering receptor expressed on myeloid cells 2 (sTREM2). These two biomarkers were selected based on their established pathological relevance to NASH progression: CK18-M30 is a well-validated circulating marker of hepatocyte apoptosis closely associated with histological disease activity (Nitze et al., 2026), and sTREM2 reflects macrophage-mediated hepatic inflammation and is widely recognized as a promising non-invasive indicator of steatohepatitis and fibrosis severity (Kothari et al., 2023). Changes in anthropometric measures: BMI, waist-to-hip ratio (WHR), and body fat percentage. Safety was assessed throughout the 12-week treatment period by monitoring adverse events, vital signs, laboratory tests (including complete blood count and renal function), and electrocardiograms.

### Sample size calculation and statistical analysis

The sample size was calculated based on the endpoint of the proportion of patients achieving a ≥30% relative reduction in liver fat content from baseline, as assessed by MRI-PDFF (Tamaki et al., 2022a). Assuming response rates of 25.9% in the placebo group (Zhao et al., 2023) and 48% in the botanical formula group after the 12-week treatment, a sample size of 99 participants per group was calculated via a two-sample test for binomial proportions, as implemented in PASS 2021 software (NCSS, LLC). This calculation was performed with a two-sided α level of 0.05% and 90% power (β = 0.10). Allowing for a 20% dropout rate, the final target enrollment was set at 248 participants (124 per group).

The primary efficacy analysis was performed using the modified intention-to-treat (mITT) population, defined as all randomized participants who received at least one dose of the study medication and had at least one evaluable post-baseline efficacy assessment. This definition is consistent with the ICH E9 guideline for superiority trials, which recommends ITT/mITT analysis as the primary approach to preserve the benefits of randomization. Of the 248 randomized participants, 20 were excluded from the mITT population because they had no evaluable post-baseline efficacy data. The reasons for the absence of all post-baseline efficacy assessments included withdrawal of informed consent (n = 5), loss to follow-up (n = 10), and treatment discontinuation due to poor adherence (n = 5). The remaining 228 participants constituted the mITT population for the primary analysis. As the mITT analysis is considered the primary and most conservative estimate of treatment effect in superiority trials, and the overall dropout rate was low and balanced between groups (8.1%, 20/248; 10 per group), no additional per-protocol sensitivity analysis was performed. For the four participants with missing post-treatment values for specific secondary outcomes (FFA, n = 1; MRI-PDFF, n = 3), multiple imputation using fully conditional specification (FCS) was applied to maximize data utilization and minimize bias. All imputation models were built under a missing at random (MAR) assumption. Each imputation model included treatment group assignment (a baseline randomization variable, unaffected by the study intervention) and the following fully observed post-treatment variables as predictors: TC, TG, HDL-C, LDL-C, BMI, WHR, and body fat percentage. These variables were selected because they are either metabolically linked to the missing outcomes or clinically correlated with hepatic steatosis severity, and all were complete in the dataset for the 228 mITT participants. Given the very low missing proportion (1.8%, 4 out of 228 mITT participants), the imputation model was parsimonious and stable. Linear regression with a normal error assumption was used as the imputation method for the continuous missing variables, and the FCS algorithm converged successfully with stable trace plots of imputed values. Five imputed datasets were generated, and results were pooled using Rubin’s rules. A sensitivity analysis comparing multiple imputation with complete-case analysis confirmed the robustness of our findings, with both analyses yielding consistent statistical significance for the primary outcome (*P* < 0.05). Categorical data are presented as frequencies and percentages. For continuous variables, normally distributed data are summarized as mean ± standard deviation, while non-normally distributed data are expressed as median with interquartile range. For the primary outcome and all secondary outcomes, inter-group comparisons were conducted as follows: independent samples t-test was used for normally distributed data (or Welch’s t-test if variances were unequal), and the Wilcoxon rank-sum test was used for data that deviated from normality. For the confirmatory analysis of the primary outcome, an analysis of covariance (ANCOVA) model was fitted, adjusting for baseline hepatic fat fraction, clinical center, and baseline BMI. The adjusted treatment effect estimate and its 95% confidence interval are reported as the primary confirmatory result, with the unadjusted between-group comparison provided as a supportive analysis.

The primary outcome, absolute change in hepatic fat fraction measured by MRI-PDFF, was the sole prespecified confirmatory endpoint. Secondary endpoints were prespecified and divided into two categories. The first category was prespecified supportive efficacy endpoints, including the proportion of patients achieving a ≥30% relative reduction in hepatic fat fraction, changes in serum liver enzymes (ALT, AST), glucose and lipid metabolism parameters (TC, TG, HDL-C, LDL-C, FFA, FBG, FINS, HOMA-IR), the validated NASH-related biomarker CK18-M30, and anthropometric measures (BMI, WHR, body fat percentage). The second category was exploratory endpoints, comprising the emerging NASH-related biomarker sTREM2 and the untargeted metabolomics analysis. Safety outcomes were prespecified for adverse event monitoring and are presented descriptively without formal hypothesis testing. No interim analyses were planned or conducted.

A two-sided *P*-value of less than 0.05 was considered statistically significant. The *P*-value for the single prespecified primary outcome is reported without adjustment for multiple comparisons. *P*-values for secondary outcomes are also presented without formal statistical adjustment, consistent with the exploratory nature of these analyses, and should be interpreted as hypothesis-generating. All baseline variables were summarized using descriptive statistics.

### Safety assessment

The safety of the HTQSHX formulation was evaluated on an *a priori* basis before trial initiation. All constituent botanical drugs are officially included in the *Chinese Pharmacopoeia* with well-established safety profiles at clinical doses, and the 24 g/day dosage was selected based on favorable tolerability data from a prior multicenter clinical trial (Zhao et al., 2012). To our knowledge, no severe adverse reactions or clinically significant drug-drug interactions have been reported for this formula at the prescribed dose. Adverse events (AEs) were monitored and recorded throughout the 12-week treatment period. Adverse drug reactions (ADRs) were defined as AEs assessed by the investigator as having a reasonable possibility of being causally related to the study intervention. All AEs underwent causality assessment by site investigators following a standardized approach, considering temporal relationship, biological plausibility, response to dechallenge (and rechallenge if applicable), and potential alternative explanations (e.g., underlying disease progression). Causality was categorized as “definitely”, “probably”, “possibly”, “unlikely”, or “unrelated”. AEs categorized as “definitely”, “probably”, or “possibly” related were classified as ADRs. Serious adverse events (SAEs) were reported to the principal investigator and the ethics committee within 24 h of awareness, in accordance with the trial protocol and regulatory requirements. This standardized causality assessment framework was applied uniformly to all adverse events, including asymptomatic serum ALT elevations, across both treatment groups.

## Results

### Participant enrollment

**FIGURE 1:**
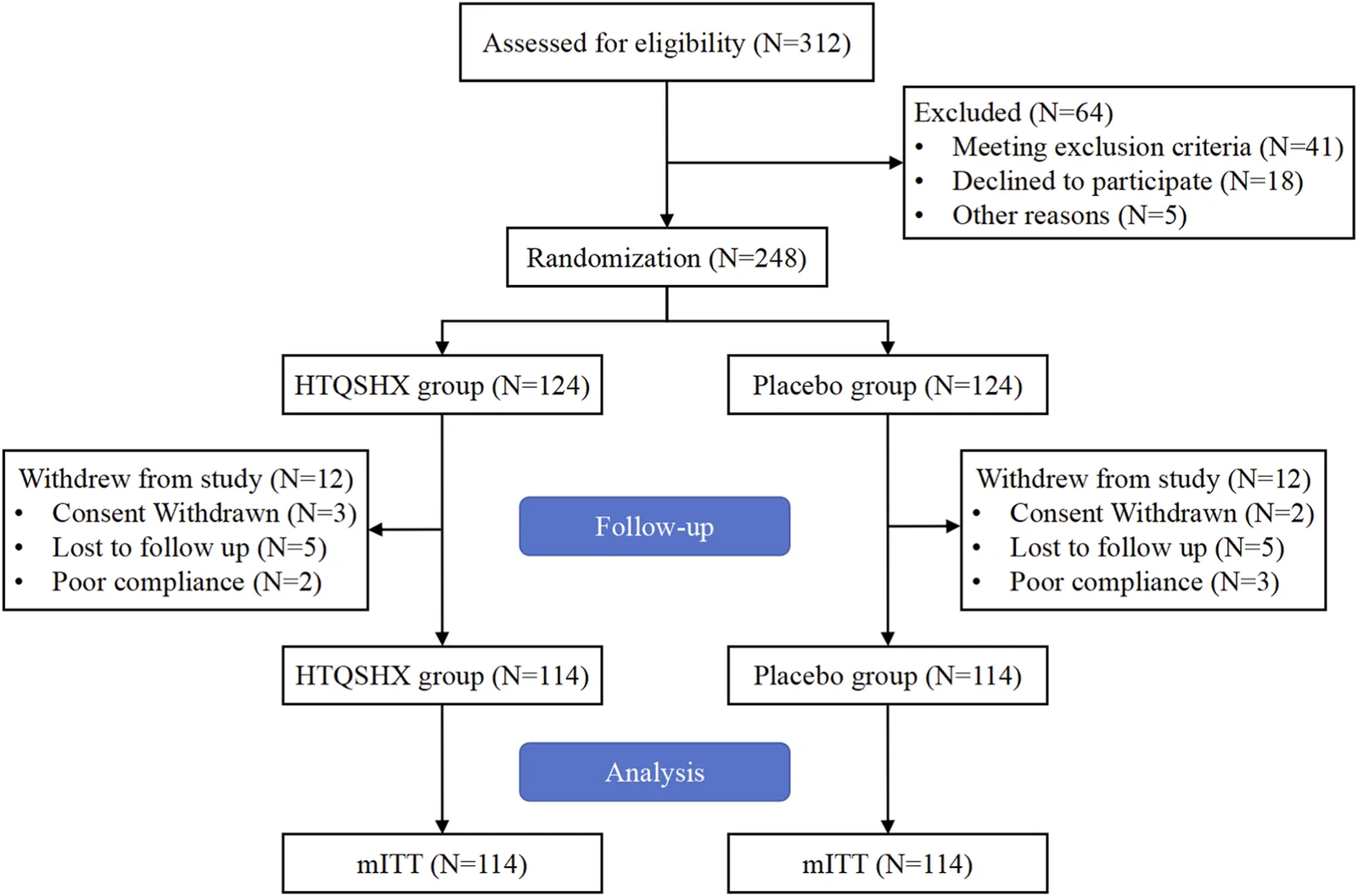
Flowchart of study on HTQSHX granules for obesity-associated non-alcoholic fatty liver disease patients. HTQSHX, Huatan Qushi Huoxue. The mITT population comprised all randomized participants who received at least one dose of the study medication and had at least one evaluable post-baseline efficacy assessment (n = 228).

Between 17 March 2023, and 11 April 2025, a total of 312 patients were assessed for eligibility at the three participating clinical centers. Of these, 64 patients were excluded during the screening phase. The primary reasons for exclusion were: meeting exclusion criteria (n = 41), declining to participate (n = 18), and other reasons (n = 5). Consequently, 248 eligible patients were enrolled and randomly assigned in a 1:1 ratio to either the HTQSHX group (n = 124) or the placebo group (n = 124). During the 12-week treatment period, 20 patients discontinued the study: 5 withdrew informed consent, 10 were lost to follow-up, and 5 discontinued the study medication due to poor adherence. Thus, 228 patients (114 in each group) completed the trial and were included in the primary mITT analysis (Figure 1). Baseline characteristics of the participants are summarized in Table 2. The two groups were generally similar with respect to demographic and clinical characteristics.

**TABLE 2:** Characteristics of the participants at baseline.

Item	HTQSHX group (n = 114)	Placebo group (n = 114)
Gender (male)	70 (61.4)	74 (64.9)
Age (year)	36 (29, 45)	37.5 (31, 44.25)
Breathing (times/min)	18 (16, 20)	18 (16, 20)
Heart rate (beats/min)	78.5 (72, 84)	78 (73, 83)
Body temperature (°C)	36.5 (36.4, 36.8)	36.6 (36.5, 36.8)
Systolic pressure (mmHg)	120 (111.5, 128)	120 (111.5, 129)
Diastolic pressure (mmHg)	77 (70, 80)	79 (70.75, 80)
Marital status (married)	83 (72.8)	88 (77.2)
Ethnicity (Han)	112 (98.2)	112 (98.2)
Occupation (non-manual workers)	93 (81.6)	91 (79.8)
Medical history (month)	21.5 (3, 60)	20 (3, 60)
Family history of NAFLD (yes)	24 (40.7)	35 (59.3)

Values are %, n (%), or median (p25, p75). HTQSHX, Huatan Qushi Huoxue; NAFLD, Non-alcoholic fatty liver disease.

### Treatment adherence

Medication adherence was high in both groups. The median adherence was 96% (IQR, 94%–98%) in the HTQSHX group and 95% (IQR, 93%–98%) in the placebo group, with no statistically significant difference between the groups (*P* = 0.101).

### Efficacy outcomes

#### Primary outcomes

**FIGURE 2:**
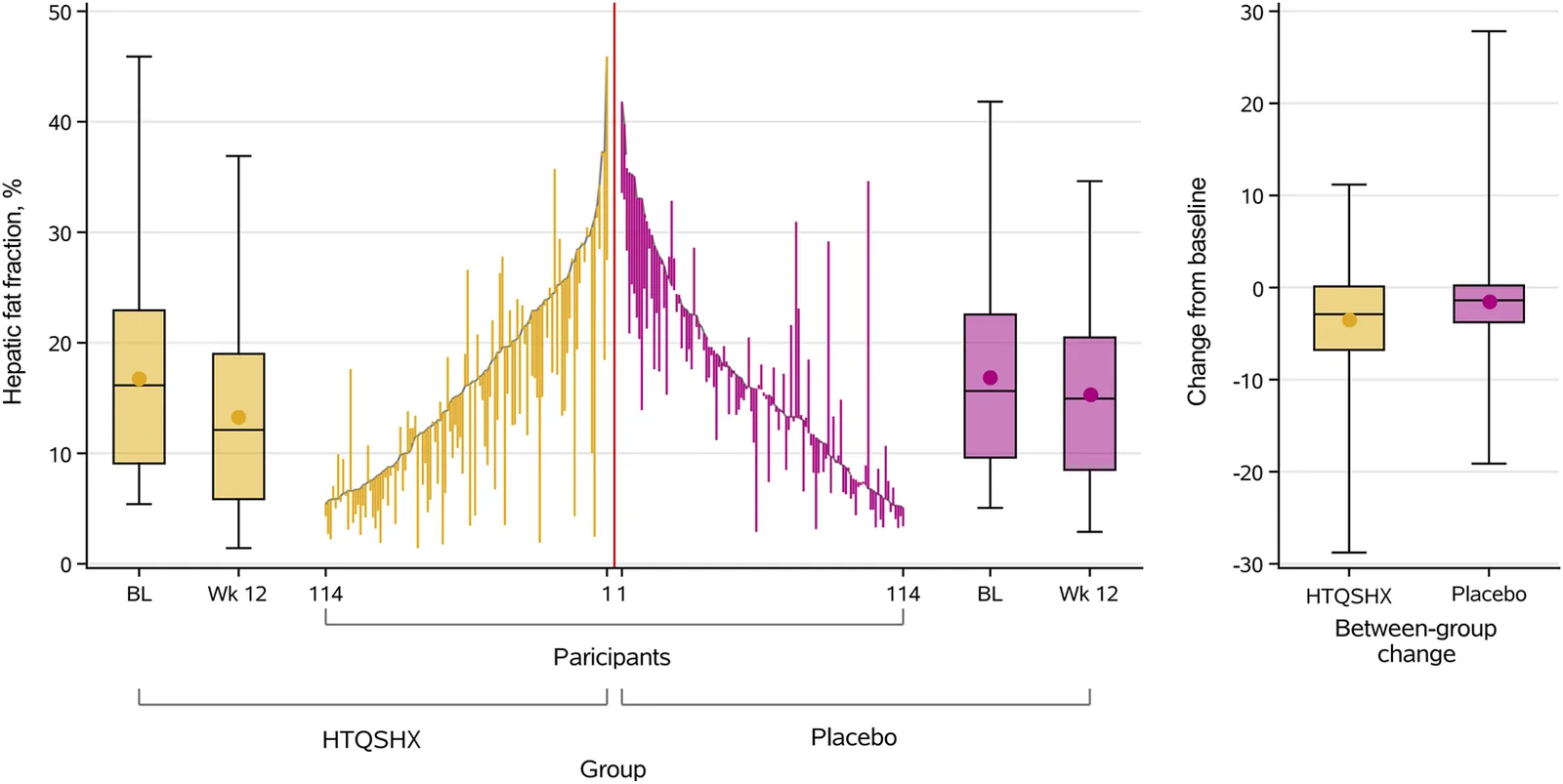
Absolute change in hepatic fat fraction by MRI-PDFF. HTQSHX, Huatan Qushi Huoxue; BL, baseline; Wk 12, Week 12.

Hepatic fat fraction was measured using MRI-PDFF. At baseline, there was no significant difference in hepatic fat content between the two groups (HTQSHX group: 16.15%, placebo group: 15.64%; *P* = 0.894). After 12 weeks of treatment, the median hepatic fat fraction decreased from 16.15% to 12.12% in the HTQSHX group and from 15.64% to 14.95% in the placebo group. Intergroup comparison revealed that the hepatic fat fraction in the HTQSHX group was significantly lower than that in the placebo group (*P* = 0.029) (*P* = 0.005 for ANCOVA adjusting for baseline) (Figure 2; Table 3).

**TABLE 3:** Changes in disease-related parameters from baseline to week 12.

Parameter	Time	HTQSHX group (n = 114)	Placebo group (n = 114)	Median difference (95% CI)	*P-*value
Primary outcome					
Absolute change in hepatic fat fraction by MRI-PDFF (%)	Baseline	16.15 (9.01, 23.03)	15.64 (9.51, 22.57)	​	0.894
​	Week 12	12.12 (5.84, 19.02)	14.95 (8.29, 20.57)	−2.32 (−4.5, −0.23)	0.029
Secondary outcomes					
Patients with a ≥30% relative decline in hepatic fat % by MRI-PDFF (n, %)	Week 12	47 (41.23%)	23 (20.18%)	​	<0.001
Serum enzymatic indicators					
ALT (U/L)	Baseline	60.9 (51, 78.25)	66.5 (52.75, 87)	​	0.179
​	Week 12	44 (29.75, 58)	48.8 (28.15, 69.9)	−6.55 (−13.60, 0.00)	0.048
AST (U/L)	Baseline	34 (25.95, 46.23)	34.9 (23.75, 46)	​	0.817
​	Week 12	26.3 (21, 33.85)	29 (22, 42.48)	−3.00 (−6.00, 0.00)	0.033
Glucose and lipid metabolism indicators					
TC (mmol/L)	Baseline	5.25 ± 0.98	5.2 ± 0.91	​	0.399
​	Week 12	5.1 ± 1.07	4.98 ± 1.03	0.08 (−0.18, 0.36)	0.828
TG (mmol/L)	Baseline	1.87 (1.36, 3.04)	1.74 (1.25, 2.39)	​	0.506
​	Week 12	1.71 (1.26, 2.78)	1.57 (1.13, 2.22)	0.20 (−0.02, 0.44)	0.071
HDL-C (mmol/L)	Baseline	1.06 (0.95, 1.27)	1.13 (1.02, 1.27)	​	0.152
​	Week 12	1.1 (0.97, 1.25)	1.13 (0.99, 1.29)	−0.03 (−0.09, 0.02)	0.260
LDL-C (mmol/L)	Baseline	3.20 ± 0.76	3.27 ± 0.72	​	0.721
​	Week 12	3.08 ± 0.79	3.13 ± 0.76	−0.06 (−0.26, 0.15)	0.835
FFA (mmol/L)	Baseline	0.57 (0.47, 0.82)	0.56 (0.41, 0.74)	​	0.206
​	Week 12	0.46 (0.33, 0.53)	0.48 (0.37, 0.62)	−0.05 (−0.09, −0.01)	0.026
FBG (mmol/L)	Baseline	5.18 (4.88, 5.58)	5.27 (4.82, 5.77)	​	0.362
​	Week 12	5.22 (4.85, 5.59)	5.22 (4.73, 5.63)	0.01 (−0.15, 0.18)	0.834
FINS (μU/mL)	Baseline	16.11 (11.30, 23.72)	15.12 (10.1, 21.51)	​	0.199
​	Week 12	13.13 (9.7, 20.75)	16.25 (10.1, 23.14)	−1.50 (−3.90, 0.80)	0.216
HOMA-IR	Baseline	3.93 (2.64, 5.72)	3.64 (2.32, 5.40)	​	0.290
​	Week 12	3.07 (2.32, 4.85)	3.76 (2.26, 5.40)	−0.31 (−0.91, 0.21)	0.251
NASH-related biomarkers					
CK18-M30 (μIU/mL)	Baseline	78.98 (56.57, 95.67)	75.16 (57.51, 95.8)	​	0.949
​	Week 12	59.49 (44.17, 73.03)	69.9 (57.7, 95.58)	−13.92 (−20.29, −7.53)	<0.001
sTREM2 (pg/mL)	Baseline	95.6 (83.86, 106.38)	98.48 (86.44, 109.84)	​	0.192
​	Week 12	84.8 (73.99, 96.13)	96.03 (83.13, 105.41)	−9.66 (−13.35, −5.79)	<0.001
Body measurement indicators					
BMI (kg/m^2^)	Baseline	28.55 (27.04, 30.82)	28.75 (26.58, 31.25)	​	0.820
​	Week 12	27.15 (25.08, 29.4)	28.05 (25.8, 30.15)	−1.00 (−1.90, 0.00)	0.046
WHR	Baseline	0.94 (0.88, 0.98)	0.94 (0.90,0.97)	​	0.998
​	Week 12	0.91 (0.85, 0.96)	0.94 (0.91, 0.97)	−0.03 (−0.05, −0.01)	0.004
Body fat percentage (%)	Baseline	31.65 (28.43, 35.6)	32.3 (27.68, 37.9)	​	0.638
​	Week 12	29.8 (26.5, 32.93)	31.6 (26.68, 37.18)	−1.80 (−3.40, −0.20)	0.024

1 Data are Mean ± standard deviation or median (IQR) unless specified.

#### Secondary outcomes

##### Patients with a ≥30% relative decline in MRI-PDFF

**FIGURE 3:**
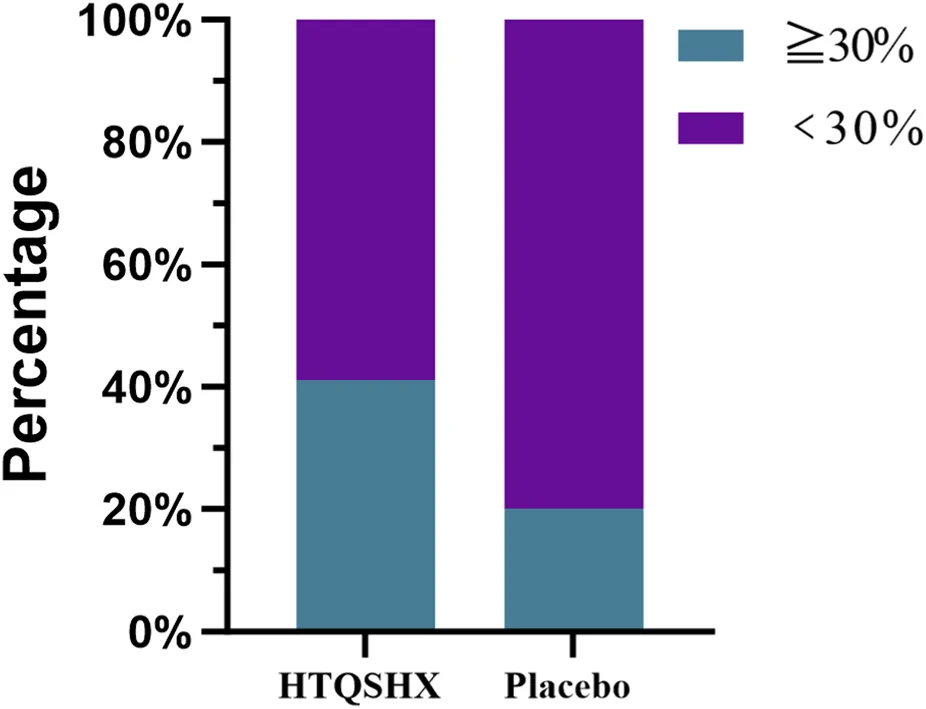
Comparison of patients with a ≥30% relative decline in hepatic fat fraction by MRI-PDFF between groups after treatment. HTQSHX, Huatan Qushi Huoxue.

After 12 weeks of treatment, 47 patients (41.23%) in the HTQSHX group achieved a ≥30% reduction in hepatic fat fraction from baseline, which was significantly higher than the 23 patients (20.18%) in the placebo group, with a statistically significant intergroup difference (*P* < 0.001) (Figure 3; Table 3).

##### Serum liver enzymes

**FIGURE 4:**
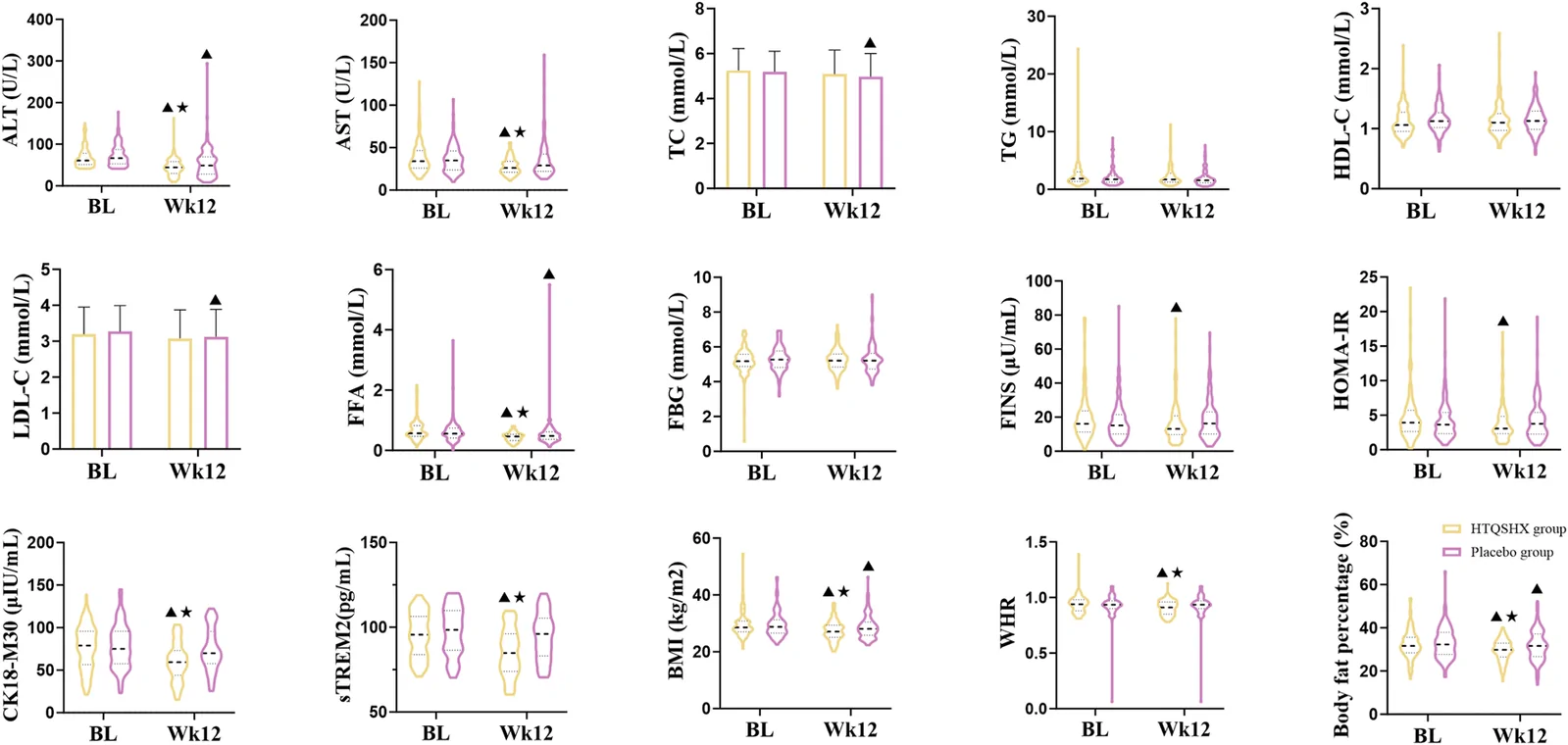
Comparison of serum liver enzymes, glucose and lipid metabolism indicators, NASH-related biomarkers, and body measurement indicators between groups before and after treatment. HTQSHX, Huatan Qushi Huoxue; BL, baseline; Wk 12, Week 12; ALT, Alanine aminotransferase; AST, aspartate aminotransferase; TC, total cholesterol; TG, triglycerides; HDL-C, high-density lipoprotein cholesterol; LDL-C, low-density lipoprotein cholesterol; FFA, free fatty acids; FBG, fasting blood glucose; FINS, fasting insulin; HOMA-IR,homeostasis model assessment of insulin resistance; CK18-M30, cytokeratin-18 fragment M30; sTREM2, soluble triggering receptor expressed on myeloid cells 2; BMI, body mass index; WHR, waist-to-hip ratio;^▲^ *P* < 0.05 vs. baseline in the same group; ^★^ *P* < 0.05 vs. Placebo group at the same time point.

Regarding ALT, the median value decreased from 60.9 U/L at baseline to 44.0 U/L in the HTQSHX group, compared to a reduction from 66.5 U/L to 48.8 U/L in the placebo group, with a statistically greater reduction observed in the HTQSHX group (*P* = 0.048). Similarly, for AST, the HTQSHX group demonstrated a more pronounced decrease from 34.0 U/L to 26.3 U/L, whereas the placebo group decreased from 34.9 U/L to 29.0 U/L (*P* = 0.033) (Figure 4; Table 3).

##### Glucose and lipid metabolism indicators

No significant intergroup differences were observed in TC, TG, HDL-C, LDL-C, FBG, FINS, or HOMA-IR between the two groups. Notably, the median FFA level decreased from 0.57 mmol/L at baseline to 0.46 mmol/L in the HTQSHX group, demonstrating a greater reduction compared to the decrease from 0.56 mmol/L to 0.48 mmol/L in the placebo group (*P* = 0.026) (Figure 4; Table 3).

##### NASH-related biomarkers

After 12 weeks of treatment, the median serum CK18-M30 level in the HTQSHX group decreased from 78.98 U/L at baseline to 59.49 U/L, which was significantly lower than the 69.9 U/L observed in the placebo group (*P* < 0.001). Similarly, serum sTREM2 showed a more pronounced reduction in the HTQSHX group, declining from 95.6 pg/mL at baseline to 84.8 pg/mL, compared to a decrease from 98.48 pg/mL to 96.03 pg/mL in the placebo group (*P* < 0.001) (Figure 4; Table 3).

##### Body measurement indicators

The median BMI in the HTQSHX group decreased from 28.55 kg/m^2^ at baseline to 27.15 kg/m^2^, while in the placebo group it decreased from 28.75 kg/m^2^ to 28.05 kg/m^2^, with between-group comparisons revealing a significantly greater reduction in the HTQSHX group (*P* = 0.046). Furthermore, the HTQSHX group demonstrated significantly greater improvements in WHR (*P* = 0.004) and body fat percentage (*P* = 0.024) compared to the placebo group (Figure 4; Table 3).

### Adverse events

No SAEs were reported during the study. Both the HTQSHX and placebo groups demonstrated favorable safety and tolerability profiles. A total of 8 AEs were reported (Table 4). Following investigator assessment of causality, 3 of these AEs were classified as ADRs. All AEs and ADRs were mild in severity. No patient discontinued the study due to an AE.

**TABLE 4:** Overview of adverse events (AEs) and adverse drug reactions (ADRs).

Item	HTQSHX group (n = 114)	Placebo group (n = 114)	*P* value
Patients with any AE, n (%)	4 (3.5%)	4 (3.5%)	1.000
Patients with any ADR, n (%)	2 (1.75%)	1 (0.88%)	1.000
Specific events (causality assessment)			
Diarrhea (assessed as possibly related, ADR)	2 (1.75%)	0	0.498
Fatigue (assessed as possibly related, ADR)	0	1 (0.88%)	1.000
Serum ALT >3×ULN (assessed as unlikely related, AE only)	2 (1.75%)	3 (2.63%)	1.000
Serious adverse events (SAEs), n (%)	0	0	—
Discontinuation due to AE, n (%)	0	0	—

In the HTQSHX group, 4 AEs occurred: two cases of diarrhea and two cases of transient, asymptomatic elevation of serum ALT >3×ULN. The diarrhea events were assessed as “possibly” related to the study drug and thus constituted ADRs. For the two cases of ALT elevation, the classification of “unlikely related” was determined according to the prespecified causality assessment criteria, based on the following integrated evidence: (a) Temporal pattern: Both elevations were detected at scheduled week 4 and week 8 visits, respectively, and resolved spontaneously without any specific intervention by the next follow-up visit, with no consistent dose-response or time-dependent worsening pattern. (b) Biological plausibility: HTQSHX granules have demonstrated consistent ALT-lowering effects in both a prior multicenter clinical trial (Zhao et al., 2012) and the overall efficacy results of the present study. A treatment-induced elevation is mechanistically inconsistent with the known pharmacological profile of the formula. (c) Absence of typical drug-induced liver injury features: No concurrent symptoms (e.g., jaundice, fatigue, nausea), no significant elevation in total bilirubin, and no eosinophilia or other signs of hypersensitivity were observed in either case. (d) Alternative explanations: Both patients had obesity-associated NAFLD with baseline ALT levels already above the upper limit of normal. The observed fluctuations fell within the range of spontaneous variability commonly seen in this patient population in the absence of active intervention. (e) Dechallenge response: ALT levels returned to near-baseline values without dose modification or study drug discontinuation. Accordingly, these events were classified as AEs rather than ADRs. The same causality assessment criteria were applied to the three cases of ALT elevation in the placebo group, which were also classified as unlikely related to the study intervention.

In the placebo group, 4 AEs occurred: one case of fatigue and three cases of serum ALT >3×ULN. The fatigue event was assessed as “possibly” related and was therefore an ADR. The ALT elevations were assessed as “unlikely related” and were not considered ADRs.

### Serum metabolomics analysis

For the exploratory untargeted metabolomics analysis, 30 subjects from the HTQSHX group were randomly selected for serum collection at baseline and post-treatment, along with 30 healthy controls for untargeted metabolomics analysis. Compared with healthy controls, the baseline group had 271 differential metabolites (*P* < 0.05), with 107 upregulated and 164 downregulated (Supplementary Material 6; Supplementary Table S2). N-fructosyl phenylalanine was markedly upregulated, whereas 1,2-dilinoleoyl-sn-glycero-3-phosphocholine, 3-alpha,20-alpha-dihydroxy-5-beta-pregnane 3-glucuronide, N-acetyl-L-methionine, and LPCs were downregulated.

Between post-treatment and baseline, 58 differential metabolites were identified (*P* < 0.05), with 27 upregulated and 31 downregulated (Supplementary Material 6, Supplementary Table S3). The post-treatment group showed significant upregulation of 2-oleoyl-1-palmitoyl-sn-glycero-3-phosphocholine, SM d34:2, PC 36:3, 1,2-dilinoleoyl-sn-glycero-3-phosphocholine, and SM d32:1.

**FIGURE 5:**
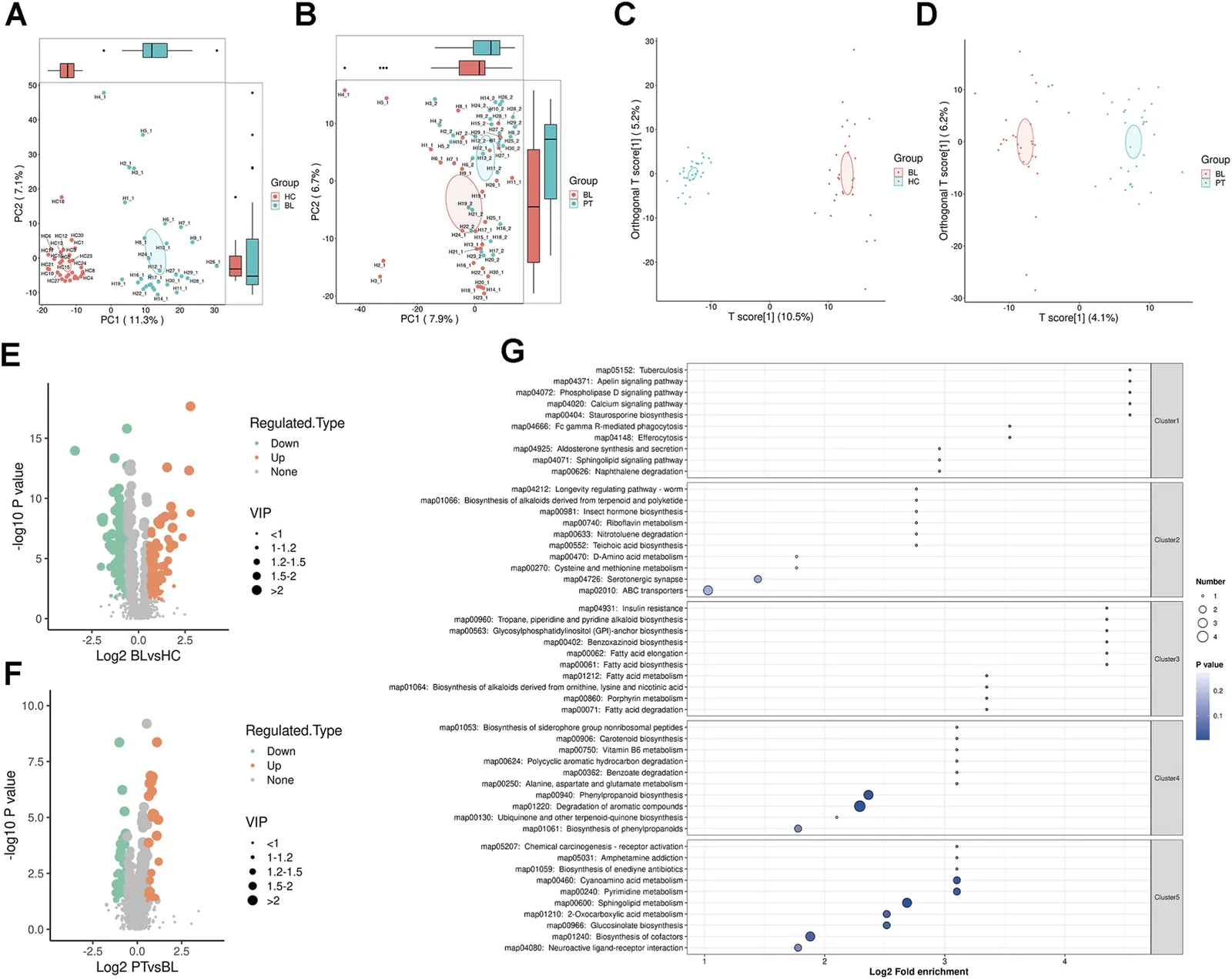
Untargeted metabolomics analysis of serum samples. HC, healthy control group; BL, baseline group; PT, post-treatment group; PCA, principal component analysis; OPLS-DA, orthogonal partial least-squares discriminant analysis. **(A,B)** PCA plots of baseline vs. healthy controls **(A)** and post-treatment vs. baseline **(B)**. In the PCA plot, each point corresponds to a sample, and the distance between two points approximately reflects the difference in metabolite composition between the two samples (Euclidean distance). **(C,D)** OPLS-DA plots of baseline vs. healthy controls **(C)** and post-treatment vs. baseline **(D)**. In the OPLS-DA plot, the horizontal axis (T) indicates the predictive metabolite score showing inter-group differences, and the vertical axis (To) indicates the orthogonal component score showing intra-group differences. Percentages denote the variance explained by each metabolite. **(E,F)** Volcano plots of baseline vs. healthy controls **(E)** and post-treatment vs. baseline **(F)**. Each point represents a metabolite. The horizontal axis shows the log_2_ fold change, and the vertical axis shows the-log_10_ (*P*-value). Green points represent downregulated metabolites, red points upregulated metabolites, and point size is proportional to the VIP value. **(G)** KEGG enrichment bubble plot of common differential metabolites among the three groups. The vertical axis lists KEGG pathways, and the horizontal axis shows the log_2_ fold enrichment. Bubble color represents the *P*-value, bubble size indicates the number of differential metabolites in the pathway, and a black border denotes *P* < 0.05.

Across the three groups, 27 differential metabolites were common (*P* < 0.05, Supplementary Material 6, Supplementary Table S4). Among these, 14 metabolites such as phosphatidylcholine PC 34:3, PC 36:3, and sphingomyelin SM d32:1 were lower in patients at baseline than in healthy controls and increased after treatment; another 8 metabolites including the oxidative stress marker 13-HODE and the mitochondrial dysfunction marker 3-hydroxybutyrylcarnitine were elevated at baseline and decreased after treatment. Enrichment analysis showed that these metabolites were primarily involved in fatty acid metabolism, sphingolipid metabolism, insulin resistance, as well as vitamin B6 metabolism, alanine/aspartate/glutamate metabolism, and D-amino acid metabolism (Figure 5).

## Discussion

Based on the core pathogenesis of “phlegm-dampness and blood stasis mutually binding in the liver collaterals” in obesity-associated NAFLD, this study evaluated the short-term therapeutic effects and safety of HTQSHX granules. The trial confirmed that this formula not only reduces hepatic fat fraction measured by MRI-PDFF but also improves liver enzymes, regulates FFA metabolism, and alleviates systemic inflammatory responses, thereby multi-targetedly intervening in the disease progression of NAFLD. This study provides reliable evidence for the clinical application of HTQSHX granules in treating NAFLD.

The HTQSHX formula is constructed based on TCM principle of compatibility (Jun-Chen-Zuo-Shi, or sovereign-minister-assistant-courier). In this formulation, Alismatis Rhizoma (Zexie) serves as the sovereign botanical drug (Jun) to promote diuresis, eliminate dampness, and reduce turbid lipids. Sargassum (Haizao) resolves phlegm and dampness, *Salvia miltiorrhiza* (Danshen) activates blood circulation and resolves stasis, and Radix Curcumae Aromaticae (Yujin) moves blood and qi; these three components jointly act as minister botanical drugs (Chen). *Crataegus pinnatifida* (Shanzha) disperses food stagnation and turbidity, *Senna tora* (L.) Roxb. (Juemingzi) clears the liver and reduces lipids, and *Silybum marianum* (Shuifeiji) clears heat and drains dampness; these three components serve as assistant botanical drugs (Zuo). Radix Bupleuri (Chaihu), the courier botanical drug (Shi), guides the actions of the formula to the liver meridian. This combination collectively aims to resolve phlegm, eliminate dampness, and activate blood circulation. HTQSHX granules contain multiple active metabolites that demonstrate multi-pathway mechanisms of action in ameliorating NAFLD and associated metabolic disorders. A randomized, double-blind, placebo-controlled crossover clinical trial indicated that daily supplementation with 500 mg quercetin for 12 weeks significantly reduced intrahepatic lipid content in NAFLD patients, an effect positively correlated with body weight reduction (Li et al., 2024). Oleanolic acid exhibits pleiotropic protective effects in animal models of obesity-associated NAFLD, markedly improving hepatic steatosis, insulin resistance, and systemic metabolic disorders (Xue et al., 2021). Tanshinone IIA alleviates hepatic steatosis, fibrosis, and inflammation in a NASH mouse model by suppressing endoplasmic reticulum stress, and modulating phospholipid metabolism (Pi et al., 2024). Luteolin ameliorates obesity-related metabolic disorders by suppressing lipogenesis, inflammation, and ectopic lipid deposition, and enhancing adipose thermogenesis and energy expenditure (Zhang et al., 2024). Although the aforementioned individual metabolites exhibit clear effects in their respective studies, the underlying mechanisms by which the formulation of HTQSHX granules exerts its overall therapeutic effect on obesity-associated NAFLD require further systematic investigation.

In this trial, MRI-PDFF was used as the primary endpoint to objectively quantify changes in hepatic fat fraction. Consistent with findings from contemporary pharmacotherapy trials, HTQSHX granules achieved a significantly greater reduction in hepatic fat fraction compared with placebo, with a median between-group difference of 2.32% in absolute change. Notably, 41.23% of patients in the HTQSHX group achieved a ≥30% relative reduction in hepatic fat fraction, a threshold associated with improved NAFLD activity score and fibrosis regression (Tamaki et al., 2022b), which was more than double the rate observed in the placebo group (20.18%). This finding confirms that HTQSHX granules effectively ameliorate hepatic steatosis, the core pathological feature of NAFLD. The clinical significance of this MRI-PDFF reduction extends beyond its statistical significance. The absolute between-group difference of 2.32% should be interpreted alongside the established surrogate endpoint framework for NAFLD: a ≥30% relative reduction in MRI-PDFF is widely validated as a surrogate for histological improvement, including a ≥2-point reduction in NAFLD activity score without fibrosis worsening (Tamaki et al., 2022b). In this trial, 41.23% of patients in the HTQSHX group achieved this clinically meaningful threshold, more than twice the 20.18% rate observed in the placebo group, providing indirect evidence of potential histological benefit. Benchmarking against published pharmacological interventions further contextualizes this effect. The 2.32% placebo-adjusted absolute reduction at 12 weeks is comparable to or exceeds the effect sizes of several established agents at longer treatment durations: for example, a 1.06% placebo-adjusted reduction in MRI-PDFF was reported with empagliflozin after 24 weeks of treatment in patients with NAFLD (Cheung et al., 2024). Concurrent improvements in liver enzymes, free fatty acids, and body composition further support the clinical relevance of these findings, reflecting multi-domain therapeutic benefits beyond isolated steatosis reduction. These data support HTQSHX granules as a promising intervention warranting further evaluation in longer-term trials with histological endpoints. While prior domestic studies have applied MRI-PDFF to evaluate acupuncture or bioactive metabolites from individual botanical drugs, this trial is, to our knowledge, the first multicenter, double-blind, placebo-controlled RCT to adopt this quantitative imaging endpoint for assessing a compound traditional Chinese botanical drug formulation in patients with obesity-associated NAFLD. This methodological rigor elevates the evidence level of TCM interventions for NAFLD and provides a reliable benchmark for future botanical formulation trials. The favorable therapeutic effects and acceptable safety profile together support a complementary role for HTQSHX granules in the current NAFLD therapeutic landscape.

HTQSHX granules were significantly superior to the placebo in reducing serum levels of both ALT and AST. The decline in transaminase levels, particularly ALT, has traditionally been regarded as a key clinical indicator of alleviated hepatocellular damage. Further analysis using serum biomarkers associated with NASH revealed that patients treated with HTQSHX granules exhibited significant reductions in both CK18-M30 and sTREM2 levels. These concordant improvements indicate that HTQSHX granules may simultaneously attenuate two core pathological processes driving NASH progression: hepatocyte apoptosis and macrophage-mediated hepatic inflammation. Collectively, these findings suggest that HTQSHX granules not only effectively reduce hepatic lipid accumulation but may also improve the overall hepatic pathological status through multiple mechanisms, including inhibiting hepatocyte apoptosis and attenuating inflammatory responses, thereby potentially conferring clinical significance in delaying the progression of NAFLD to NASH and liver fibrosis. HTQSHX granules significantly reduced serum free fatty acid (FFA) levels compared to placebo. As a central pathophysiological factor in NAFLD progression, elevated FFA not only provides substrates for hepatic triglyceride synthesis but also induces lipotoxicity by triggering endoplasmic reticulum stress, mitochondrial dysfunction, and inflammatory activation. This reduction in FFA suggests that HTQSHX granules may alleviate hepatic lipid overflow and lipotoxicity, potentially through suppressing peripheral lipolysis and/or promoting hepatic FFA oxidation. Moreover, HTQSHX treatment produced greater improvements in BMI, waist-to-hip ratio, and body fat percentage, indicating beneficial effects on central obesity and fat distribution. This overall therapeutic effect aligns with the multi-target and holistic regulatory characteristics of traditional Chinese medicine formulations.

HTQSHX granules exhibited a favorable safety profile during the 12-week treatment, which is consistent with the *a priori* safety assessment of the formulation. All eight constituent botanical drugs have a long history of clinical use in China and well-documented safety records in the *Chinese Pharmacopoeia*. The 24 g/day daily dose was selected based on favorable tolerability data from a prior multicenter clinical trial (Zhao et al., 2012). In the present study, the overall incidence of adverse events was comparable between groups, consisting primarily of mild, transient gastrointestinal reactions such as diarrhea. No serious adverse events were reported, and no patient discontinued the study due to an adverse event. Collectively, these findings support a favorable benefit-risk profile for HTQSHX granules and justify further clinical development of the formulation.

Notably, despite significant reductions in hepatic steatosis and inflammatory biomarkers, several systemic metabolic parameters (HOMA-IR, fasting glucose, fasting insulin, total cholesterol, LDL-C) showed no significant between-group differences. This dissociation is well recognized in NAFLD intervention trials and does not contradict the proposed metabolic mechanism of HTQSHX granules. First, hepatic responses consistently precede systemic metabolic improvements, as NAFLD represents the hepatic manifestation of whole-body metabolic dysfunction rather than its primary driver. The significant reduction in circulating free fatty acids, a core mediator of hepatic lipotoxicity, provides direct evidence that HTQSHX granules already modulate the key metabolic pathway underlying hepatic steatosis. Second, the 12-week intervention is sufficient to detect robust changes in hepatic fat fraction via the highly sensitive MRI-PDFF assay, but relatively short to remodel systemic metabolic homeostasis driven by multifactorial processes across adipose tissue, muscle, and pancreas. Uniform lifestyle counseling provided to all participants further narrowed between-group differences in these systemic endpoints. Third, concurrent improvements in body composition (BMI, waist-to-hip ratio, body fat percentage) and metabolomic evidence of restored phospholipid metabolism and reduced oxidative stress indicate ongoing metabolic benefits that have not yet translated to statistically significant changes in routine clinical parameters. Future studies with longer treatment duration and enrollment of patients with more pronounced baseline metabolic dysregulation are warranted to fully characterize the systemic metabolic effects of the formula.

Our metabolomics analysis revealed that obesity-associated NAFLD patients exhibited a distinct metabolic disorder characterized by elevated N-fructosyl phenylalanine and reduced levels of multiple phospholipids including LPCs, PC 34:3, and PC 36:3. After 12 weeks of HTQSHX treatment, 27 metabolites were significantly regulated. Notably, beneficial phospholipids such as PC 36:3 and SM d32:1 were restored, while the oxidative stress marker 13-HODE and mitochondrial dysfunction marker 3-hydroxybutyrylcarnitine were decreased. Enrichment analysis showed that these metabolites were primarily involved in fatty acid metabolism, sphingolipid metabolism, and insulin resistance pathways, along with vitamin B6 and amino acid metabolism. These findings suggest that HTQSHX granules ameliorate NAFLD through multi-target regulation of lipid homeostasis, oxidative stress, and mitochondrial function. These metabolomic changes are consistent with the significant reductions in hepatic fat fraction, serum ALT/AST, and FFA levels observed in the HTQSHX group, providing mechanistic support for the therapeutic effects of the granules observed in this study.

## Limitations and future perspectives

This study has several limitations that should be acknowledged. First, the 12-week intervention duration is sufficient to detect changes in the surrogate endpoint of hepatic fat fraction, but is inadequate to evaluate long-term histological outcomes such as fibrosis progression. Second, instead of liver biopsy, the histological reference standard, we used internationally validated non-invasive imaging and serum biomarkers for efficacy assessment, which prevents direct correlation of our findings with histopathological endpoints. Third, while the primary outcome was prespecified and statistically powered, the multiple secondary endpoints were analyzed without formal adjustment for multiple comparisons, which carries a risk of type I error; these secondary findings should therefore be interpreted as hypothesis-generating. Fourth, the untargeted metabolomics analysis included only the HTQSHX group and healthy controls, without a parallel placebo arm. This design precludes definitive attribution of observed metabolic changes to the study intervention alone, as contributions from lifestyle modification or natural biological variation cannot be fully excluded. This analysis was designated as exploratory from the outset, constrained by pragmatic considerations of analytical cost and sample availability. Fifth, all enrolled patients had obesity-associated NAFLD, so the findings cannot be directly generalized to non-obese NAFLD populations with distinct pathophysiological features. Finally, the multi-metabolite mechanisms of HTQSHX granules have only been preliminarily supported by clinical and metabolomic evidence; a more systematic elucidation of its active metabolites and pharmacodynamic basis remains needed.

Looking ahead, several research directions are warranted. Longer-term, larger-scale trials with histological endpoints are required to confirm the sustained efficacy of HTQSHX granules and to evaluate its effects on fibrosis progression. Future studies should also include metabolomic profiling in both treatment arms to validate the exploratory metabolic findings and to definitively establish treatment-specific mechanistic effects. Trials enrolling broader patient populations, including non-obese patients with NAFLD and patients with more pronounced metabolic dysregulation, will help clarify the generalizability and optimal target population of this formulation. Finally, deeper pharmacological research into the active metabolites and core targets of HTQSHX granules will be valuable to strengthen the mechanistic evidence base for its clinical application.

## Conclusion

The findings of this study demonstrate that HTQSHX granules produce significantly greater effects than placebo in treating obese patients with NAFLD and exhibit a favorable safety profile. This trial provides high-level evidence-based support for HTQSHX granules as a potential adjunctive therapy in the comprehensive management of obesity-associated NAFLD, with important practical implications for expanding clinical options and improving metabolic outcomes in this patient population. Metabolomics analysis further reveals that HTQSHX granules restore phospholipid metabolism and reduce oxidative stress, offering mechanistic insights into its therapeutic effects.

## Data Availability

The original contributions presented in the study are included in the article/Supplementary Material, further inquiries can be directed to the corresponding author.
