# Supplementary material for: Effects and safety of Huatan Qushi Huoxue granules on obesity-associated non-alcoholic fatty liver disease: a multicenter, randomized, double-blind, placebo-controlled trial
Source: Front Pharmacol. 2026 Aug 25;17:1899068. doi: 10.3389/fphar.2026.1899068 (PMC13550174; doi:10.3389/fphar.2026.1899068)
Supplement: Supplementary file 1 [file Table1.DOCX]

Number ：AF/SC-08/03.2

**Ethical Review Consent Letter**

| Opinion Number | 2022HL-038-01 | | |
| --- | --- | --- | --- |
| Project Name | Efficacy and safety of Huatan Qushi Huoxue granules on obesity-associated non-alcoholic fatty liver disease: a multicenter, randomized, double-blind, placebo-controlled trial | | |
| Project Source | Henan Province's "double first-class" creation of scientific research in traditional Chinese medicine | | |
| research unit | The First Affiliated Hospital of Henan University of Chinese Medicine (leading unit); Shuguang Hospital Affiliated to Shanghai University of Traditional Chinese Medicine (participating unit), Shaanxi Provincial Hospital of Traditional Chinese Medicine (participating unit) | | |
| Principal Investigator | Wenxiao Zhao, Xiaoyan Liu, Minghao Liu, Chenxiao Wang, Lihui Zhang, Haowei Liang | | |
| Review category | reexamination | Review method | Quick review |
| Review date | March 3rd, 2022 | Inspection site | No. 19 Renmin Road, Zhengzhou City, Henan Province |
| **review documents** | 1. Application for Reexamination  2. Revised informed consent form (version number: 01, version date: December 31, 2021)  3. Accept the application form for ethics review of multicenter research (applicable to multicenter research, our institution is a participating unit)  4. Clinical study protocol (version number: 01, version date: December 30, 2021)  5. Materials for recruiting subjects (version number: 01, version date: December 30, 2021)  6. Case Report Form (Version Number: 01, Version Date: December 30, 2021)  7. Researcher's Handbook  8. Professional resume of the main researcher  9. GCP or research ethics training certificate for research team members  10. Research project approval/task book (cover page and funding page) | | |
| **Examination Opinion** | | | |
| According to the ethical principles of the "Ethical Review Measures for Biomedical Research Involving Human Subjects" (2016) issued by the National Health Commission, the "Good Clinical Practice for Drugs (2020)" and "Good Clinical Practice for Medical Devices (2016)" issued by the National Medical Products Administration and the National Health Commission, the "Helsinki Declaration" issued by WMA, and the "International Ethical Guidelines for Biomedical Research on Human Subjects" issued by CIOMS, after review by this ethics committee, we agree to conduct this study in accordance with the above clinical research protocol, informed consent form, and recruitment materials.  Please follow the GCP principles and adhere to the protocol approved by the ethics committee to conduct clinical research and protect the health and rights of participants.  Prospective, interventional clinical studies that complete clinical trial registration on publicly accessible databases before recruiting the first subject.  If there is a change in the main researcher during the research process, any modifications to the clinical research protocol, informed consent form, recruitment materials, etc., please submit an amendment review application.  Serious adverse events have occurred, please submit a serious adverse event report in a timely manner.  Please submit a research progress report one month before the deadline in accordance with the annual/regular follow-up review frequency specified by the ethics committee; The applicant shall submit a summary report of the research progress of each center to the ethics committee of the leading unit; When there are any situations that may significantly affect the conduct of the experiment or increase the risk to the subjects, the applicant is requested to submit a written report to the ethics committee in a timely manner.  The study included subjects who did not meet the inclusion criteria or exclusion criteria, met the termination criteria but did not allow subjects to withdraw from the study, received incorrect treatment or dosage, or received concomitant medications prohibited by the protocol, among other situations where the study was not conducted in accordance with the protocol; Or situations that may have adverse effects on the rights/health of the subjects and the scientific validity of the research that violate the GCP principles, please  The applicant/monitor/researcher submits a violation report of the protocol.  If the applicant suspends or terminates the clinical study in advance, please submit a report on the suspension/termination of the study in a timely manner.  Complete clinical research, please submit a research completion report by the applicant.  This clinical trial shall be implemented within one year from the date of consent. If it is not implemented within the time limit, this consent letter shall be automatically invalidated. | | | |

| Annual/Regular Tracking Review Frequency | 12 months |
| --- | --- |
| Next tracking review deadline | March 7th, 2023 |
| Validity period | 2022-03-08~2023-03-07 |
| Contact person and phone number | 0371-66285929 |
| Signature of Chairman/Vice Chairman | Chunfang Wang |
| Ethics Committee | Ethics Committee of the First Affiliated Hospital of Henan University of Traditional Chinese Medicine |
| Date | March 8th, 2022 |
